# Adult Day Service Use Decreases Likelihood of Depressive Symptoms Among Black Dementia Caregivers

**DOI:** 10.1093/geroni/igaa057.2102

**Published:** 2020-12-16

**Authors:** Lauren Parker, Laura Gitlin

**Affiliations:** 1 Johns Hopkins Medicine, Baltimore, Maryland, United States; 2 Drexel University, Philadelphia, Pennsylvania, United States

## Abstract

Black Americans are more likely than others to age with Alzheimer’s Disease (AD) in the community and rely on family members for support. Despite reported positive aspects of caregiving, Black caregivers report greater need for daytime respite and caregiving support. Little is known regarding the health-promoting benefits of daytime respite, like adult day services (ADS), among Black caregivers. Using a sample of 190 Philadelphia-area Black caregivers for community-living persons with dementia, pooled from two behavioral intervention trials: Advancing Caregiver Training and Care of Persons with Dementia in their Environments, the study examined the association between ADS use and depressive symptoms. About 36% of the caregivers used adult day services for their family member with AD. Controlling for demographic variables, social support, self-rated health, religious coping, caregiver burden, and number of years caregiving Black caregivers who utilized ADS had lower depressive symptoms (β= -1.60, p<.05) relative not using ADS.

